# Association between estimated pulse wave velocity and silent lacunar infarct in a Korean population

**DOI:** 10.3389/fcvm.2023.1070997

**Published:** 2023-01-25

**Authors:** Yaping Zhou, Yu Zhang, Gang Xu, Xiuli Shang

**Affiliations:** ^1^Department of Rehabilitation Medicine, Shanghai Tenth People’s Hospital, Tongji University School of Medicine, Shanghai, China; ^2^Department of Neurology, The First Hospital of China Medical University, Shenyang, China

**Keywords:** arterial stiffness, estimated pulse wave velocity, silent lacunar infarct, cerebral small vessel disease, Korean population, aging

## Abstract

**Aims:**

Previous studies have proposed the estimated pulse wave velocity (ePWV) as a simple and cost-effective measure of arterial stiffness. Since arterial stiffness plays a role in the progression of silent lacunar infarct (SLI), our present work aims to evaluate the association between ePWV and the presence of SLI.

**Methods:**

The present work was based on a cross-section study. Our study included 1,011 neurologically healthy Korean participants. The SLI was evaluated using brain magnetic resonance images (MRI). The ePWV was derived from a published equation using age and mean blood pressure (MBP). Logistic regression analyses were performed to investigate the association between ePWV and SLI. The linear relationship and robustness were evaluated using smooth curve fitting and subgroup analyses, respectively.

**Results:**

The prevalence of SLI was 11.87%. After fully adjusting for covariates, per 1 m/s increase of ePWV casted 31% additional risk for SLI (*P* = 0.009). When dividing the ePWV into quartiles, the top quartile had 4.01 times risk compared with the bottom quartile. The increasing trend across the quartiles was statistically significant (*P* for trend < 0.001). Consistently, smooth curve fitting revealed that the risk of SLI elevated linearly with the increase of ePWV. Finally, subgroup analysis suggested that the association was robust in several sub-populations divided by age, sex, smoking, hypertension, diabetes mellitus (DM), coronary artery occlusive disease (CAOD), hyperlipidemia, and statin medication (all *P* for interaction > 0.05).

**Conclusion:**

The current study revealed an independent and positive association between ePWV and the presence of SLI in a neurologically healthy Korean population.

## 1. Introduction

Arterial stiffness is a typical vascular structural and functional alteration increased with age and characterized by decreased vascular distensibility and compliance (1). Increased arterial stiffness is associated with an increased risk of cardiovascular and cerebrovascular diseases (2–7), including hypertension, diabetes, chronic kidney disease, cardiovascular disease (CVD), coronary heart disease, heart failure, stroke, and death. Identifying arterial stiffness effectively may help refine risk stratification and prevent cardiovascular and cerebrovascular diseases.

Measurement of pulse wave velocity (PWV) is a representative technique for assessing arterial stiffness (8), with carotid-femoral pulse wave velocity (cfPWV) regarded as the gold-standard measurement (9). However, since measurements of PWV rely on trained expertise and specialized devices, the measured PWV is not widely applicable in clinical practice, especially in primary healthcare hospitals. Under this circumstance, the estimated pulse wave velocity (ePWV) was proposed as a simple surrogate to indicate arterial stiffness, with higher ePWV levels suggesting a stiffer artery (10). The ePWV was calculated simply from age and mean blood pressure (MBP) and has been proven to have a close association with measured cfPWV (10). Later studies have verified the reliability of ePWV to indicate aortic stiffness and serve as a risk indicator in several CVDs (10, 11). Moreover, alterations in vessel wall stiffness captured by ePWV may reflect systemic arteriosclerosis from fibrosis and calcification and thus parallel lesion development in the cerebrovasculature (12–14). However, whether the risk-indicating capacity of ePWV could be generalized in cerebral vascular diseases associated with arterial stiffness has yet to be tested.

Silent lacunar infarct (SLI) is one of the most common subtypes of cerebral small vessel diseases (15). Although SLI is a silent event (16), SLI is closely related to an increased risk of future symptomatic stroke, dementia, psychiatric disorders, and early mortality (17–19). Considering its clinical silence (16), high prevalence (20–22), and adverse impacts (17–19), better recognition of the risk indicators and adequate prevention of SLI are imperative. Pathological research suggested that arterial stiffness contributed to the development of SLI (23, 24). However, previous reports about the association between SLI and measured PWV biomarkers were inconsistent (25–27), and whether ePWV is correlated with the presence of SLI was undetermined. Therefore, the present study aims to investigate the association between ePWV and the presence of SLI.

## 2. Materials and methods

### 2.1. Data source and participants

The present work is a secondary analysis. All the data used in the current work originated from Lee et al.’s article (28), a published open-access article. The data was also used in a secondary study by Yao et al. (29) published in *Frontiers in Neurology*. Lee et al.’s research (28) was a cross-sectional study based on a Korean population. The authors extracted data from a database from March 2008 to December 2014. A total of 1,441 patients were included according to the inclusion criteria: (1) neurologically healthy individuals with underlying cardiovascular risk factors or a family history of stroke, (2) aged ≥45 years, and (3) individuals who have undergone magnetic resonance imaging (MRI) and magnetic resonance angiography (MRA) scans of the brain. A total of 430 individuals were excluded for the following reasons: (1) inadequate medical information (*n* = 93); (2) no laboratory tests performed (*n* = 154); (3) no data on brain MRI or MRA (*n* = 84); (4) previous history of neurological disease (*n* = 39); (5) abnormal neurological findings at the time of examination (*n* = 28); and (6) history of liver disease, including active hepatitis, liver cirrhosis, and hepatoma (*n* = 32). Finally, 1,011 participants were included.

The study protocol of Lee et al.’s research (28) was approved by the Institutional Review Board (IRB) of CHA Bundang Medical Center (IRB No. BD-2010083). Consequently, the present study did not need to be reviewed by a local Ethics Committee since it was an analysis based on Lee et al.’s work. All subjects provided informed consent.

### 2.2. Data collection and measurement

In the current analyses, we selected covariates that could impact the association between ePWV and SLI, including age, gender, smoking, diastolic blood pressure (DBP), systolic blood pressure (SBP), total cholesterol, triglyceride, fasting plasma glucose (FPG), estimated glomerular filtration rate (eGFR), hypertension, diabetes mellitus (DM), coronary artery occlusive disease (CAOD), hyperlipidemia, and statin medication. Smoking refers to having smoked within 1 year before attending (28). Hypertension The eGFR was calculated according to the abbreviated Modification of Diet in Renal Disease Study Equation (30). Hypertension was determined if SBP ≥ 140 mmHg, DBP ≥ 90 mmHg, or on anti-hypertension therapy (28). DM was diagnosed if the patient was on anti-diabetes therapy or FPG ≥ 126 mg/dL (28). Patients with CAOD refer to those with a history of acute myocardial infarction or unstable angina or were diagnosed with CAOD by auxiliary examinations (28). Hypercholesterolemia was defined as total cholesterol being ≥240 mg/dL or on lipid-lowering medication (28).

As previously reported (28), MRI scans were obtained using a 1.5 T MR system. A neurologist and a radiologist assessed all the images for the presence of SLI, blinded to the clinical and laboratory information of participants. SLI was defined as small (3–15 mm in diameter) lesions in the deep perforating arteries supplied area that shows low signal intensity on T1-weighted images and high signal intensity on T2-weighted and fluid-attenuation inversion recovery (FLAIR) images (28, 31).

The ePWV was calculated according to the following equation: ePWV = 9.587 −0.402 × age + 4.560 × 10^–3^ × age^2^ −2.621 × 10^–5^ × age^2^ × MBP + 3.176 × 10^–3^ × age × MBP −1.832 × 10^–2^ × MBP (10). The MBP was determined as DBP + 1/3 (SBP−DBP).

### 2.3. Statistical analysis

According to the presence of SLI, all participants were divided into two groups (with or without SLI). Continuous variates were expressed as mean ± standard deviation (SD, normal distribution) or median (quartile 1- quartile 3, skewed distribution). Categorical variables were summarized as frequency (percentage). The Student’s *t*-test (or Mann–Whitney test) and the Chi-square (χ^2^) test (or Fisher’s exact tests) were introduced to compare continuous and categorical variates between two groups, respectively. We performed multivariate logistic regression analyses to explore the relationship between ePWV and the presence of SLI. The covariates in multivariate logistic regression models were selected following three criteria: First, variates that have been identified as risk factors for SLI; Second, variates that were recognized as factors associated with SLI in previous studies; Third, variates that showed significant association with SLI or ePWV in preliminary univariate regression analyses. The results were expressed as odds ratio (OR) and 95% confidence interval (CI). Furthermore, our study conducted a generalized additive model with a smooth spline function to explore the linear relationship between ePWV and the presence of SLI. Finally, subgroup analyses were introduced in several subgroups to assess the reliability and robustness of the association between ePWV and SLI.

All the statistical analyses were performed by SPSS 26.0 software (IBM corp, Chicago, IL, USA), statistical software packages R (The R Foundation),^1^ and EmpowerStats (X&Y Solutions, Inc., Boston, MA, USA).^2^ Statistical significance was determined as a two-tailed *P*-value < 0.05.

## 3. Results

### 3.1. Baseline characteristics of participants

In the present study, 1,011 participants were included. We summarized the baseline characteristics of the participants in Table 1. The prevalence of SLI is 11.87%. People with SLI showed a higher level of age, SBP, and MBP (all *P*-values < 0.05). The prevalence of hypertension and DM was significantly higher in the SLI population (all *P*-values < 0.05). Moreover, compared with people without SLI, those who suffered from SLI showed a lower level of eGFR (*P* = 0.012). Most importantly, the ePWV level of the SLI population was significantly higher compared with that in the group without SLI (*P* < 0.001).

**TABLE 1:** Characteristics of participants.

Characteristics	Total (*N* = 1,011)	Without SLI (*N* = 891)	With SLI (*N* = 120)	*P*-value
Age, years	64.2 ± 9.1	63.6 ± 9.1	68.4 ± 8.2	<0.001
Sex (male, %)	359 (35.5)	305 (34.2)	54 (45.0)	0.021
Smoking (%)	205 (20.3)	175 (19.6)	30 (25.0)	0.17
SBP (mmHg)	131.7 ± 18.3	130.8 ± 17.4	138.3 ± 22.9	<0.001
DBP (mmHg)	80.0 ± 11.5	79.9 ± 11.4	81.4 ± 12.6	0.165
MBP (mmHg)	97.3 ± 12.5	96.8 ± 12.2	100.4 ± 14.4	0.004
Fasting glucose (mg/dL)	108.0 (95.0–146.5)	108.0 (95.0–149.5)	110.0 (94.8–141.2)	0.598
Total cholesterol (mg/dL)	193.8 ± 39.8	193.8 ± 39.4	194.2 ± 43.1	0.929
Triglyceride (mg/dL)	127.0 (88.0–180.5)	126.0 (88.0–177.5)	131.0 (84.8–197.5)	0.062
Hypertension (%)	579 (57.3)	480 (53.9)	99 (82.5)	<0.001
Diabetes mellitus (%)	224 (22.2)	185 (20.8)	39 (32.5)	0.004
Hyperlipidemia (%)	332 (32.8)	292 (32.8)	40 (33.3)	0.902
CAOD (%)	52 (5.1)	44 (4.9)	8 (6.7)	0.421
Statin medication (%)	227 (22.5)	201 (22.6)	26 (21.7)	0.826
eGFR (mL/min/1.73 m^2^)	74.1 ± 16.8	74.6 ± 16.6	70.5 ± 18.1	0.012
ePWV (m/s)	10.3 ± 1.8	10.1 ± 1.8	11.2 ± 1.7	<0.001

CAOD, coronary artery occlusive disease; ePWV, estimated pulse wave velocity; eGFR, estimated glomerular filtration rate; SLI, silent lacunar infarct.

### 3.2. Association between ePWV and SLI

A significant and independent association between ePWV and SLI was observed in the multivariate logistic regression analyses (Table 2). In the crude model, per 1 m/s increase of ePWV leaded to 1.37 times risk of SLI (*P* < 0.001). When adjusting for covariates including age, sex, smoking, DM, CAOD, hyperlipidemia, statin medication, fasting glucose, and eGFR, per 1 m/s increase of ePWV still caused 31% additional risk of SLI (*P* = 0.009). When dividing the ePWV into quartiles, the top quartile was 4.01 times more likely to have SLI compared with the bottom quartile after full adjustment for potential confounding. The increasing trend across the quartiles was statistically significant (*P* < 0.001).

**TABLE 2:** Multivariable logistic regression analysis of ePWV for the presence of SLI.

	OR (95% CI)					
	Crude	*P*-value	Model I	*P*-value	Model II	*P*-value
ePWV (m/s)	1.37 (1.24, 1.53)	<0.001	1.29 (1.06, 1.57)	0.012	1.31 (1.07, 1.60)	0.009
**Quartiles of ePWV**						
Quartiles 1	Reference	–	Reference	–	Reference	–
Quartiles 2	1.09 (0.51, 2.30)	0.831	0.93 (0.43, 2.05)	0.866	0.99 (0.46, 2.11)	0.976
Quartiles 3	2.98 (1.57, 5.64)	<0.001	2.38 (1.07, 5.29)	0.033	2.70 (1.40, 5.19)	0.003
Quartiles 4	4.48 (2.42, 8.32)	<0.001	3.11 (1.19, 8.16)	0.021	4.01 (2.12, 7.60)	<0.001
*P* for trend	–	<0.001	–	0.006	–	<0.001

Crude: no adjustment; Model 1: adjusted for age, sex; Model 2: adjusted for age, sex, smoking, DM, CAOD, hyperlipidemia, statin medication, fasting glucose and eGFR. CAOD, coronary artery occlusive disease; CI, confidence interval; DM, diabetes mellitus; ePWV, estimated pulse wave velocity; eGFR, estimated glomerular filtration rate; OR, odds ratio; SLI, silent lacunar infarct.

### 3.3. Linear relationship between ePWV and SLI

**FIGURE 1:**
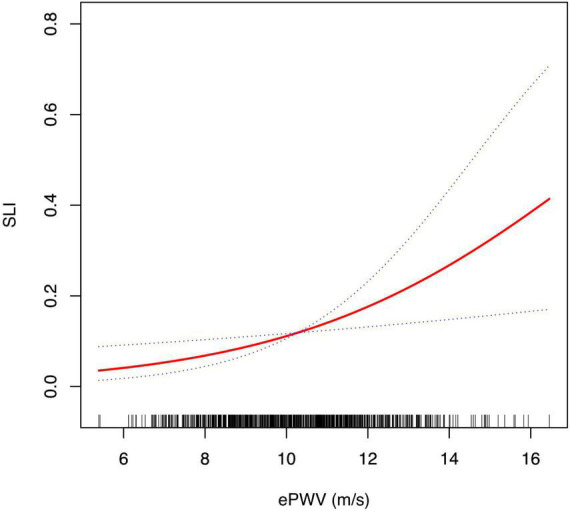
Smoothing curve fitting of the association between ePWV and the risk of SLI. Smooth curve fitting was performed using a generalized additive model to determine the correlation between ePWV and the risk of SLI after adjusting for age, sex, smoking, DM, CAOD, hyperlipidemia, statin medication, fasting glucose and eGFR. In this figure, the solid red line represents the estimated risk of SLI, and the dotted blue lines represent a point wise 95% confidence interval. CAOD, coronary artery occlusive disease; DM, diabetes mellitus; ePWV, estimated pulse wave velocity; eGFR, estimated glomerular filtration rate; SLI, silent lacunar infarct.

Smooth curve fitting with a generalized additive model suggested the association between ePWV and SLI was linear in the whole range of ePWV (Figure 1). The result was in accordance with the significant increase trend observed in the multivariate logistic regression model.

### 3.4. Subgroup analysis

**FIGURE 2:**
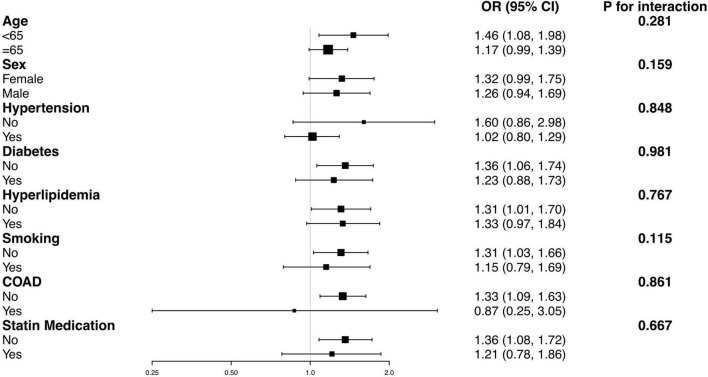
Subgroup analysis for the impact of ePWV on the SLI. The dots and lines indicate the estimates of the odds ratios of SLI for ePWV and the corresponding 95% confidence intervals, respectively. The multivariate logistic model adjusted for age, sex, smoking, DM, CAOD, hyperlipidemia, statin medication, fasting glucose and eGFR, except for the variable that is stratified. None of the stratified variables significantly modified the impact of ePWV on the risk of SLI (all *P* for interaction > 0.05). CAOD, coronary artery occlusive disease; DM, diabetes mellitus; ePWV, estimated pulse wave velocity; eGFR, estimated glomerular filtration rate; SLI, silent lacunar infarct.

We divided the participants into groups according to age, sex, hypertension, DM, hyperlipidemia, smoking, CAOD, and statin medication. Multivariate logistic regression analyses were re-conducted in these subgroups, with all the covariates used in Table 2 adjusted, except for the variate used for stratification. The results indicated a consistent relationship between ePWV and SLI in all the subgroups (all *P* for interaction > 0.05). The results are exhibited in Figure 2.

## 4. Discussion

The present study evaluated the relationship between ePWV and the presence of SLI. The findings showed an independent and positive association between ePWV and the risk of prevalent SLI. Furthermore, a linear relationship existed in the whole range of ePWV. Moreover, the results suggested that the correlation was robust in several specific sub-populations divided by cardiovascular risk factors, including age, sex, smoking, hypertension, DM, hyperlipidemia, CAOD, and statin medication. Our results supported the association between arterial stiffness and the presence of SLI.

The ePWV is a newly proposed index to reflect the severity of arterial stiffness, calculated from an equation combining age and MBP (10). Later studies proved that the ePWV was reliable in indicating arterial stiffness and closely associated with measured cfPWV (10, 11). Since the ePWV is simple and easily accessible, ePWV may serve as an initial screening measure for risk prediction, especially in primary healthcare conditions (32). Several subsequent research proved the capacity of ePWV to predict the risk of hypertension, cardiovascular outcomes, and all-cause mortality, independent of traditional CVD risk factors (10, 14, 33–35). Moreover, ePWV was also reported to be associated with non-cardiovascular mortality and residual-specific mortality (11, 36). However, the risk stratification capacity of ePWV for cerebral vascular diseases has yet to be tested.

Silent lacunar infarct is one of the most prevalent subtypes of cerebral small vascular disease, with a high prevalence range from 10–20% in the general population to 55% in clinical-based healthy patients (20, 21). In disease-specific populations, the prevalence climbs to higher than 90% (22). SLI is usually found incidentally on computed tomography (CT) or brain MRI, especially in elderly patients with hypertension and DM (16). Due to its clinically asymptomatic, SLI may often not get enough attention (17). However, SLI contributes to multiple symptomatic neurological diseases. SLI in basal ganglia could damage local and remote white matter integrity, result in cognition impairments, and increase the risk of dementia (17, 37). Compared with those without SLI, stroke-free people with SLI showed a more than twofold risk of future first symptomatic stroke (18). Moreover, SLI could independently raise the risk of recurrent stroke in patients with a first-stroke history (38). Furthermore, SLI was also associated with psychiatric disorders and all-cause mortality (19, 39). Considering the clinical silence, high prevalence, and adverse impacts, finding a simple and effective risk indicator may help to improve early recognition, prevention, and burden-relieving of SLI.

Arterial stiffness may contribute to the development of SLI. Arterial stiffness damages the compliance of the vessel wall, resulting in increased pulse pressure and systemic hypertensive impairments (23, 24). Greater arterial stiffness leads to increased transmission of pulsatile pressure into the microcirculation, increasing cerebral small vessel damage (40). Moreover, due to low vascular resistance, cerebral arterials are highly vulnerable to pulsatile systemic pressure (24). Therefore, arterial stiffness could be a potential risk factor for SLI. Since ePWV is a surrogate of arterial stiffness and exhibited risk indication capacity in multiple diseases related to arterial stiffness, we hypothesized that ePWV may be associated with SLI.

The results confirmed our hypothesis. The multivariate logistic regression analyses implicated a positive and independent correlation between ePWV and the presence of SLI after adjusting for cardiovascular risk factors, suggesting the potential value of ePWV to identify the risk of SLI. The results also showed a significant increasing tendency between the prevalence of SLI and the increment of ePWV. Therefore, we introduced the smooth curve fitting to evaluate the relationship between ePWV and SLI. The result showed a linear association between ePWV and the risk of prevalent SLI in the whole range of ePWV, which was consistent with the significant trend observed in the multivariate logistic regression models. Furthermore, we performed subgroup analysis to test the robustness of the association in some common sub-populations. The results confirmed a robust relationship between ePWV and SLI in subpopulations divided by age, sex, hypertension, DM, hyperlipidemia, smoking, CAOD, and statin medication (all *P* for interaction > 0.05).

Previous reports about the association between arterial stiffness biomarkers and SLI were inconsistent (25–27). Henskens et al. (25) reported that, in 167 hypertensive patients, a 1-SD increase in measured aortic PWV was independently associated with a 78% increment of risk in the presence of lacunar infarcts. Consistently, Kim et al.’s study (26), which recruited 1,282 patients with acute ischemic stroke or transient ischemic attack, showed that brachial-ankle PWV (baPWV) was associated with chronic lacunes independently, with per 1-SD increase in baPWV casted 1.24-times risk of chronic lacunes. While on the contrary, Matsumoto et al. (27) measured baPWV in 480 asymptomatic voluntary participants. The results showed that the association between baPWV and silent cerebral infarction was only found in univariate analysis but not in multivariate analysis after adjusting cardiovascular risk factors (27). The inconsistency in results may be due to population differences. Our study supported the findings of an association between arterial stiffness and SLI in a neurological healthy population with underlying cardiovascular risk factors or a family history of stroke. Large sample size and population-based studies are needed to further demonstrate the association between ePWV and SLI in the future.

The present study still has some limitations. Firstly, the original cohort study on which our study based was not designed to study arterial stiffness, which may cause some bias to the results. A more exhaustive protocol designed to study arterial stiffness and involving cerebral arterial territories will make our results more rigorous. Although our current work revealed an independent association between ePWV and the presence of SLI, the causal relationship between ePWV and the progression of SLI cannot be determined since the present analysis is based on a cross-section study. Longitudinal prospective research is needed in the future to unfold causal relationships between ePWV and the presence of SLI. Secondly, the participants in our study were neurological healthy Korean population with underlying cardiovascular risk factors or a family history of stroke. Consequently, whether the present findings can be generated for the other population remains to be determined. Thirdly and lastly, because the current work was a secondary analysis, some variates were inaccessible due to being unrecorded in the original study. As with other observational studies, unrecorded variates may lead to residual confounding and bring bias into the analysis accordingly. Therefore, large studies with more detailed information collection are needed to verify the relationship between ePWV and SLI in the future.

## 5. Conclusion

In summary, the present study demonstrated an independent and linear association between ePWV and the presence of SLI. Elevated ePWV was significantly correlated with increased risk of SLI. The results supported an association between arterial stiffness and SLI. Moreover, our results suggested that the association was robust in several common subpopulations divided by cardiovascular risk factors.

## Data availability statement

The original contributions presented in this study are included in the article/supplementary material, further inquiries can be directed to the corresponding authors.

## Ethics statement

The studies involving human participants were reviewed and approved by the Institutional Review Board (IRB) of CHA Bundang Medical Center (IRB No. BD-2010-083). The patients/participants provided their written informed consent to participate in this study.

## Author contributions

YaZ contributed to the manuscript drafting, data analysis, and interpretation. YuZ contributed to the manuscript drafting. XS and GX proposed the concept of the manuscript. All authors have read and approved the final manuscript.

## Footnotes

1
http://www.R-project.org

2
http://www.empowerstats.com
